# Oxygen-doped carbon nanotubes for near-infrared fluorescent labels and imaging probes

**DOI:** 10.1038/s41598-018-24399-8

**Published:** 2018-04-19

**Authors:** Yoko Iizumi, Masako Yudasaka, Jaeho Kim, Hajime Sakakita, Tsukasa Takeuchi, Toshiya Okazaki

**Affiliations:** 1 CNT-Application Research Center, National Institute of Advanced Industrial Science and Technology (AIST), 1-1-1 Higashi, Tsukuba, 305-8565 Japan; 2 Nanomaterials Research Institute, National Institute of Advanced Industrial Science and Technology (AIST), 1-1-1 Higashi, Tsukuba, 305-8565 Japan; 3 Electronics and Photonics Research Institute, National Institute of Advanced Industrial Science and Technology (AIST), 1-1-1 Umezono, Tsukuba, 305-8568 Japan; 4 Shimadzu Corporation, 1-3 Kanda Nishiki-cho Chiyoda, Tokyo, 101-8448 Japan

## Abstract

Chemical modification of carbon nanotube surface can controllably modulate their optical properties. Here we report a simple and effective synthesis method of oxygen-doped single-walled carbon nanotubes (o-SWCNTs), in which a thin film of SWCNTs is just irradiated under the UV light for a few minutes in air. By using this method, the epoxide-type oxygen-adducts (ep-SWCNTs) were produced in addition to the ether-type oxygen-adducts (eth-SWCNTs). The Treated (6, 5) ep-SWCNTs show a red-shifted luminescence at ~1280 nm, which corresponds to the most transparent regions for bio-materials. Immunoassay, fluorescence vascular angiography and observation of the intestinal contractile activity of mice were demonstrated by using the produced o-SWCNTs as infrared fluorescent labels and imaging agents.

## Introduction

Since single-walled carbon nanotubes (SWCNTs) have diameters of 1–2 nm and lengths of several micrometers, and their optical properties show unique one-dimensional aspects^1^ such as optical transitions between van Hove singularities^2^, large exciton effects^3^ and length-dependent plasmon absorptions^4^. In particular, the photoluminescence (PL) in the near-infrared (NIR) region, which is related to the optical transitions between the first band gap of semiconducting SWCNTs (*E*_11_), has been expected for bio-applications because the emission range of about 900–1400 nm is ideal for biological imaging due to the low tissue autofluorescence and low absorption coefficient of water^5,6^.

It has recently been reported that modest chemical functionalization can control the optical properties of carbon materials^7–11^. With regards to SWCNTs, the introduction of sp^3^ defects into sp^2^ carbon networks can create a new optically allowed defect state. Consequently, the PL is red-shifted and can be over 10 times brighter^10,11^. For example, covalent doping of the nanotube surface with a low concentration of oxygen atoms is an effective method of controlling nanotube optical properties^10,12,13^.

Oxygen-doped SWCNTs (o-SWCNTs) were first prepared by the exposure of ozone to the SWCNT solution under ultra-violet (UV) light^10^. Because very low ozone doses are required to prepare such o-SWCNTs, prolonged exposure from hours to days is necessary to control the doping reaction^10,12,13^. A comparison of the experimental observations with the calculated energy shifts suggests that the ether-type oxygen adducts (eth-SWCNTs) are mainly produced with this solution method^10,13^. The eth-SWCNTs can also be fabricated by photoreaction with diphenyl disulfide in the presence of oxygen^14^. The doping reaction is induced by electron transfer from excited SWCNTs to disulfide, which requires UV irradiation of around 6 h^14^. Solid-state approaches for producing o-SWCNTs have recently been reported^15,16^. The oxide dielectric film coating of SWCNTs on various substrates effectively lead to oxygen doping^15,16^. The o-SWCNTs fabricated with this method show two PL peaks, indicating that the epoxide-type oxygen adducts (ep-SWCNTs) are produced as well as eth-SWCNTs^15,16^. Although this method provides an easy route toward fabrication of photostable o-SWCNTs in solid-state matrices, it may not be easy to fabricate them in large quantities.

We here report on a simple and efficient fabrication method of o-SWCNTs, with which the SWCNT thin film is exposed to ozone by using a conventional UV ozone cleaner for a few minutes. The fabricated o-SWCNTs show additional NIR fluorescence at a longer wavelength than that of eth-SWCNTs^13^. Based on the PL energy shift of ~300 meV after the reaction, the peak was reasonably assigned to that of ep-SWCNTs. The (6, 5) ep-SWCNTs showed emission at ~1280 nm, which is particularly advantageous for NIR imaging. Ghosh *et al*. reported that o-SWCNTs increase the image contrast in living cells due to the increase in detected signal and decrease in autofluorescence, when compared with the pristine nanotubes^13^. However, biosensing and bioimaging applications of o-SWCNTs have so far been limited. Hence, bio-medical applications by using o-SWCNTs as NIR fluorescent labels for immunoassay and NIR fluorescent probes for *in vivo* imaging of mice are demonstrated.

## Results and Discussion

**Figure 1:**
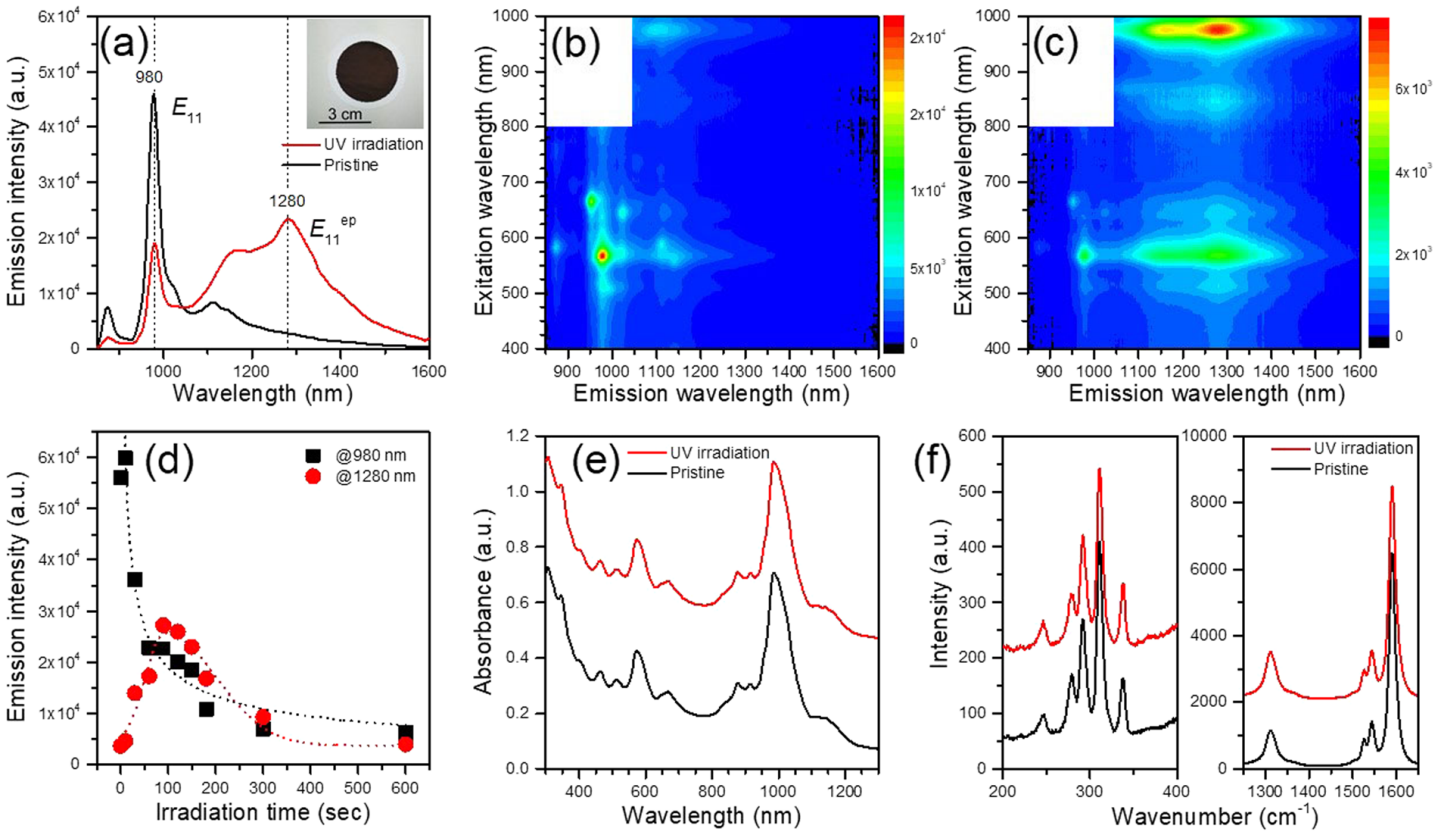
Spectral changes of SWCNTs after exposure to UV-ozone process. (**a**) PL spectrum obtained from SDBS-D_2_O solution of SWCNTs after UV irradiation for 90 sec (red line), together with reference spectrum of pristine SWCNT-SDBS-D_2_O solution (black line). Excitation wavelength = 570 nm. (inset) Image of treated SWCNT thin film. (**b**) 2D excitation-emission contour plots of (**b**) pristine and (**c**) treated SWCNTs. (**d**) UV-irradiation time dependence of PL intensities of *E*_11_ at 980 nm (black) and that of epoxide adducts at 1280 nm (E_11_^ep^) (red). (**e**) Absorption spectrum of processed SWCNTs after 90-sec UV irradiations (red), together with reference spectrum of original SWCNTs (black). (**f**) Resonance Raman spectrum of treated SWCNTs after 90-sec irradiations (red), together with reference spectrum of pristine SWCNTs (black).

Oxygen doping in SWCNTs was conducted using UV irradiation with a conventional UV ozone cleaner (Meiwafosis, PC-450 plus). Briefly, 1.0 mg of a (6, 5)-SWCNTs enriched sample (Aldrich, 773735 Carbon nanotube, single-walled) was dispersed in ethanol (10 ml) using a bath-type sonicator for 10 min, and the solution was filtered. After drying the SWCNT thin film on a membrane, SWCNTs were irradiated with a UV light for 90 sec with the UV ozone cleaner. The UV intensity was ~19 mW/cm^2^ at the sample position. Figure 1(a) shows the PL spectrum obtained from the dodecylbenzene sulfonate (SDBS, Aldrich)-D_2_O solution of o-SWCNTs (red line), together with the reference spectrum of the pristine SWCNT-SDBS-D_2_O solution (black line). The excitation wavelength was 570 nm, which corresponds to the *E*_22_ transition of (6, 5) SWCNTs. In the PL spectrum of the pristine SWCNTs, intense emission at around 980 nm was clearly observed, which can be attributed to the *E*_11_ exciton emission of the (6, 5) SWCNTs^17^. However, the spectral shape drastically changed after UV irradiation. A new peak was observed at ~1280 nm with a shoulder at 1160 nm, while the original *E*_11_ emission at 980 nm diminished. The latter PL at 1160 nm can attributed to eth-SWCNTs^10^.

The PL features are clearer in the two-dimensional (2D) excitation-emission contour plots of the pristine and treated SWCNTs shown in Fig. 1(b) and (c), respectively. In the PL contour plot of the pristine SWCNTs (Fig. 1(b)), there is an intense PL peak at 980 nm in emission and 570 nm in excitation, which corresponds to the *E*_11_ excitonic emission of (6, 5) SWCNTs. On the other hand, a new emission at ~1280 nm was observed at the same *E*_22_ excitation wavelength of 570 nm for the treated SWCNTs (Fig. 1(c)), suggesting that the emission was red-shifted upon UV irradiation. Because the absorption coefficient of *E*_11_ is greater than that of *E*_22_, the emission intensity at the excitation wavelength of 980 nm is larger than that of 570 nm^10^.

The energy difference between the new and original PL emissions is ~304 meV, which is very close to that of ep-SWCNTs reported previously^13^. Ma *et al*. observed that oxygen-doping caused a spectral shift of 300 meV for (6, 5) SWCNTs^13^. Based on the density functional theory (DFT) calculations, they concluded that the shift is induced by the epoxide adducts. Ohfuchi also reported that the formation of epoxide-type o-SWCNTs is energetically reasonable for (6, 5) SWCNTs from the DFT calculations^18^. Almost the same value of the present energy shift strongly suggests that the ep-SWCNTs were fabricated with our fabrication method. The PL intensity ratio between eth-SWCNTs and ep-SWCNTs was 1:1.3 based on the spectral fitting result (see Supporting Information).

The oxygen-doping reaction in the present study was faster than that of the solution processes^10,12–14^. Figure 1(d) shows the UV-irradiation time dependence of the PL intensities of *E*_11_ at 980 nm (black) and that of the epoxide adducts at 1280 nm (*E*_11_^ep^) (red). During irradiation of UV light, the intensity of *E*_11_^ep^ emission increased, whereas that of *E*_11_ emission decreased until ~90 sec. Given that the reaction time in the solution methods are hours to days^10,12–14^, the present reaction is, at least, two orders of magnitude faster.

The shape of the absorption spectrum remained unchanged upon UV irradiation. Figure 1(e) shows the absorption spectrum of the fabricated SWCNTs after 90-sec UV irradiations, together with a reference spectrum of the original SWCNTs. Note that the absorption peaks at ~1280 nm and ~1160 nm are barely observable in the spectrum of the modified SWCNTs. Almost the same spectral shapes suggest that the amount of oxygen-doped sites is quite low, as is the case of o-SWCNTs reported previously^10^.

The reaction with the ozone was accompanied by an increase in the D-band, signifying covalent functionalization of the nanotube sidewall. Figure 1(f) shows the resonance Raman spectrum of the treated SWCNTs after 90-sec irradiations, together with the reference spectrum of the pristine SWCNTs. The integrated intensity ratio of the D-band (~1310 cm^−1^) and G-band (1500–1600 cm^−1^) increased after UV irradiation (D/G = 0.33), compared with that of the pristine SWCNTs (D/G = 0.20). Similar distributions of the radial breathing mode (230–340 cm^−1^) between the treated o-SWCNTs and original SWCNTs indicates that the oxygen-substituting reaction occurs irrespective of the chiral indices of SWCNTs.

SWCNTs is considered one of the most promising candidates for NIR fluorophores^19–31^ because of several advantages such as wide emission ranges depending on the SWCNT structures^17^, large gap between excitation and emission wavelengths^17^, and high photo-stability^32^. Fortunately, the emission wavelength of (6, 5) ep-SWCNTs was ~1280 nm, which is located at the most transparent region of biomaterials, the so-called second-window NIR (NIR-II)^5,6^. Hence, we demonstrated the bio-applications of o-SWCNTs to NIR fluorescent labels for immunoassay and NIR fluorescent probes for *in vivo* imaging.

**Figure 2:**
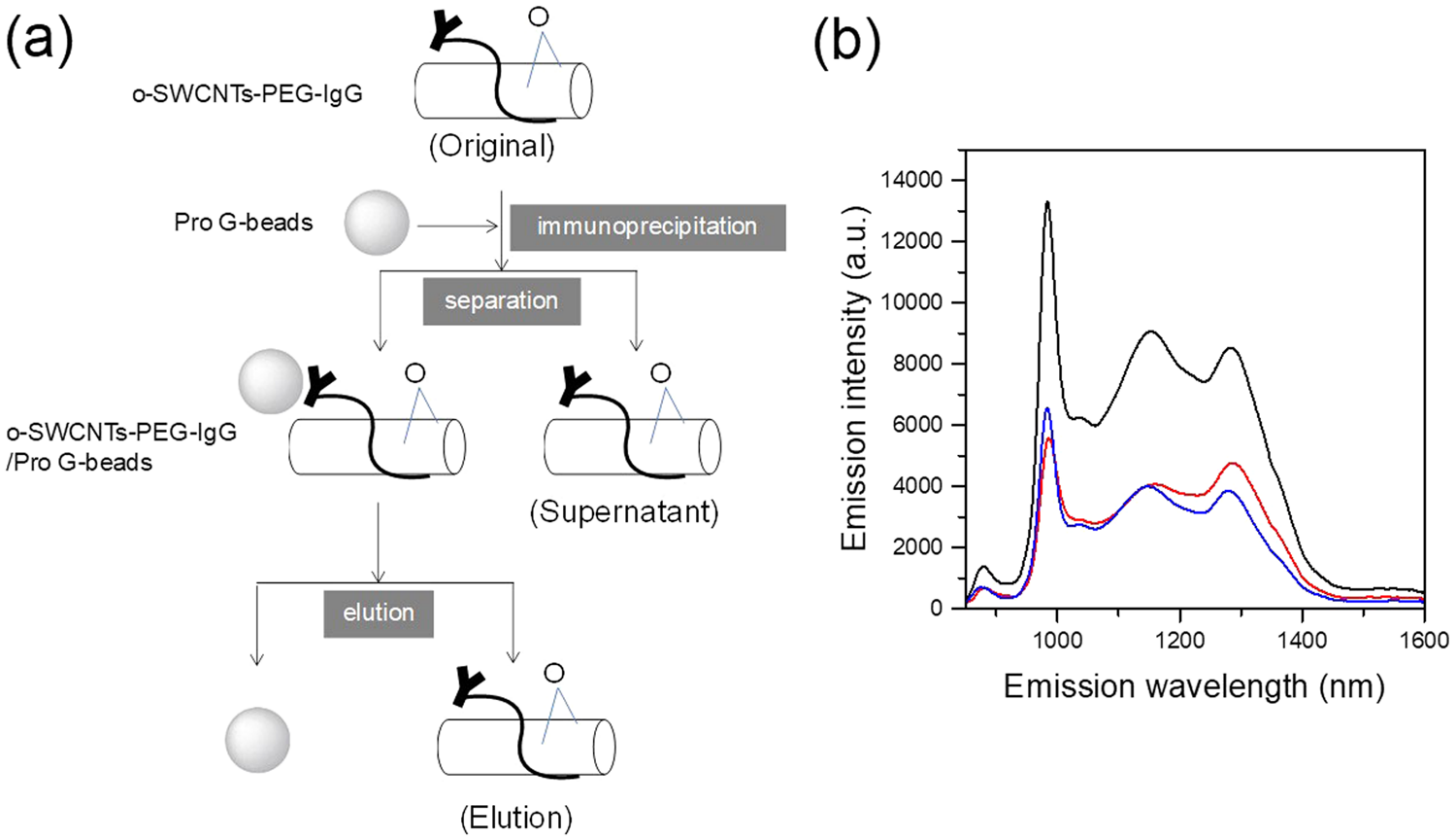
(**a**) Flow chart of our immunoassay experiment. (**b**) PL spectra of original (black), supernatant (red), and elution (blue) of o-SWCNTs. Excitation wavelength = 570 nm.

Figure 2(a) shows the flow chart of our immunoassay experiment. First, the o-SWCNTs were coated with N-(Methylpolyoxyethylene oxycarbonyl)-1,2-distearoyl-sn-glycero-3-phosphoethanolamine conjugate immunogloburin G (PEG-IgG) by dialyzing the SDBS solution of o-SWCNTs (see Methods). The black line in Fig. 2(b) shows the PL spectrum obtained from the o-SWCNT-PEG-IgG solution. Note that the optical absorption of water drastically diminishes the PL of o-SWCNTs beyond 1300 nm (see Fig. 1(a)). Immunoprecipitation (IP) of o-SWCNT-PEG-IgGs were carried out with protein G-attached magnetic beads (Pro G-beads) (Fig. 2(a)). After the IP reaction, the o-SWCNT-PEG-IgGs were collected using a permanent magnet and eluted from the beads. The characteristic PL signal of the o-SWCNTs was successfully observed from the elution (blue line in Fig. 2(b)). Notably, the sum of the PL intensities of the elusion and supernatant was almost the same as that of the original o-SWCNT-PEG-IgG solution. This coincidence means that quantitative analysis is possible by using o-SWCNTs as fluorescent labels, similar to pristine SWCNTs^27^.

**Figure 3:**
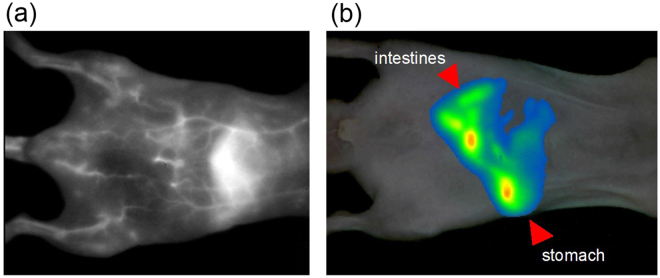
(**a**) NIR fluorescence image of mouse intravenously injected with o-SWCNTs. (**b**) Overlays of white light and NIR fluorescence images of mouse 5 min after oral administration of o-SWCNTs.

Next, we discuss *in vivo* NIR imaging of mice by using o-SWCNTs as fluorescent probes. Figure 3(a) shows the *in vivo* angiography for a live mouse 20 min after injection of the o-SWCNTs-PEG solution into the tail vain. The excitation wavelength was tuned to an *E*_11_ peak of (6, 5) SWCNTs (=980 nm). The PL signals below ~1080 nm were cut by using a long pass filter. An extremely clear image of o-SWCNTs circulating in the vasculature under the skin was obtained. Because o-SWCNTs can flow in capillaries, the entire body of the mouse was imaged. The high special resolution was consistent with the fact that the wavelength around 1300 nm showed a much lower scattering coefficient than the other wavelength^24,25^. Reduced photon scattering in this spectral region allows fluorescence imaging into a deep region of blood vessels with sufficient spatial resolution. Due to the high spatial resolution in the depth direction, the blood vessels near the epidermis and liver are spatially distinguishable from each other.

Due to the high intensity of the fabricated nanotubes, non-invasive NIR fluorescence imaging of the murine digestive organs was successfully observed through oral administration. Figure 3(b) shows overlays of white light and NIR fluorescence images of a mouse 5 min after oral treatment. Because o-SWCNTs can exhibit enough emission intensity even at low pH, the shape of the stomach could be clearly seen as well as that of the intestine. As shown in Videos S1–S3 (Supporting Information), the motion of the digestive organs, such as intestinal contractile activity, was imaged with sufficient temporal resolution. Such intestinal transit and contractility are important parameters in gastrointestinal (GI) motility studies^33^. Videos S1–S3 suggest that o-SWCNTs can provide information about murine intestinal transit and contractility in a fast, accurate, and easy-to-implement manner. The SWCNT fluorescence imaging and spatio-temporal motility mapping may facilitate the study of GI motility under both normal and disease conditions.

## Conclusion

We reported on a simple and efficient fabrication method of o-SWCNTs. Ultra-violet irradiation on the SWCNT thin film with a conventional UV-ozone cleaner led to sufficient amounts of o-SWCNTs for a few minutes. Epoxide-type oxygen adducts were fabricated with our method. The emission wavelength of the (6, 5) ep-SWCNTs was located at 1280 nm in aqueous solution, which is the most transparent wavelength region of bio-materials. Immunoassay and *in vivo* imaging of mice were successfully demonstrated using o-SWCNTs as NIR imaging labels and probes. In addition to NIR fluorescent labels and probes, o-SWCNTs are also useful for photonic materials due to the oxygen-induced deep trap states^15,16^. For instance, Ma *et al*. successfully carried out fluctuation-free, room-temperature single-photon emission in the telecom wavelength range of 1300–1500 nm by using ep-SWCNTs^15^. With our fabrication method, researchers can easily obtain o-SWCNTs to study the basic properties of SWCNTs and their bio- and photonic applications.

## Methods

### Spectroscopy

For the PL and optical absorption measurements, micelle solutions of the o-SWCNT samples were prepared. Briefly, 1.0 mg of the treated o-SWCNT sample was dispersed in ~10 mL of D_2_O containing 1 wt% of SDBS (Aldrich) using a 200 W tip-type sonicator (SONICS, VCX500) equipped with a titanium alloy tip (TI-6AL-4V). Typical sonication time was 10 min. The solution was then centrifuged at 175000 g for 1 h (HITACHI-Koki, CS100GXII) to remove bundle forms of o-SWCNTs, after which the supernatant was obtained. The total amounts of the o-SWCNTs in the collected supernatant was estimated to be ~50% by the optical absorption spectroscopy. The PL measurements were carried out with a NIR fluorescence spectrophotometer system using an InGaAs array detector (Horiba Jobin-Yvon, Fluorolog-3-2-iHR320). Optical absorption spectra were recorded with a Shimadzu UV-3100 spectrometer. The concentration of ep-SWCNTs was estimated using the integrated absorption intensity from 800 to 1250 nm.

For Raman spectroscopy, the o-SWCNT micelle solution was filtered to form an o-SWCNT thin film, and the thin film was transferred onto high-resistance silicon wafers, which were grown using the floating zone method. The resonance Raman spectra were measured using a triple-grating T64000 monochromator system (Horiba Jobin-Yvon) with diode-pumped solid-state lasers (Spectra Physics) (532 nm).

### Preparation of o-SWCNTs fluorescent labels and imaging probes

Biocompatible aqueous solutions of o-SWCNTs were prepared for IP and *in vivo* imaging. First, we dispersed o-SWCNTs into water with SDBS by the same procedure as for the spectroscopic measurements (see above). The o-SWCNTs-SDBS solution was diluted with 1 wt% of SDBS solution to make a concentration of 50 μg/ml. For IP, the 400 μl of ep-SWCNTs-SDBS solution was filtered using centrifugal filtration devices (Omega Pall Corporation Nanosep, MWCO 300 K) at 12000 rpm for 10 min, and 400 μl of a phosphate buffer solution (PB) (50 mM, pH 6.2) containing 0.1 wt% of PEG (NOF Corporation SUNBRIGHT DSPE-050CN) was added and centrifuged. The SDBS was replaced with PEG in this washing process. After three repetitions of the washing process, the micellar solution of o-SWCNTs (o-SWCNT-PEG) was prepared. For *in vivo* imaging of the mice, 3 mg of PEG was added to the 1 ml of o-SWCNT-SDBS solution and dissolved with a bath-type sonicator for 3 min. The resultant mixture was then dialyzed with a 3,500 molecular weight cut-off membrane (Spectrum, Float-A-Lyzer G2) for 3 days. The dialysis outer liquid was changed to new water several times per day. This process slowly replaced the PEG from the SDBS. We measured the absorption spectrum of the displaced water to check the residual concentration of SDBS. After 3 days, more than 95% of SDBS was replaced.

### Immunoprecipitation

For preparation of o-SWCNT-PEG-IgGs, the o-SWCNT-PEGs in PB solution (216 μl) was mixed with 24 μl rabbit IgG solution (Aldrich, I5006, 5 mg/ml) and left out at 5 °C overnight for the reaction. After the reaction, the excess IgG was removed by flushing the PB dispersion of o-SWCNT-PEG-IgGs through a column (Clontech, CHROMA SPIN + TE-200 columns). The obtained solution was referred to as the “original” solution. In the IP experiment of the o-SWCNT-PEG-IgGs, the protein-G-coated magnetic beads (ProG-beads) (Ademtech, Bio-Adembeads Protein G, 40 μl) were used. According to the manufacturer’s instruction, ProG-beads were washed twice with and dispersed in 40 μl of Dulbecco’s phosphate-buffered saline (−) (Wako) containing 0.5 v% of Tween20 (Wako) before IP. Then, the o-SWCNT-PEG-IgG solution (40 μl) was mixed with ProG-beads collected with a permanent magnet at room temperature and left at 4 °C for about 1 h. After the IP treatment, 40 μl of the dispersion of the o-SWCNT-PEG-IgG/ProG-beads was collected with a permanent magnet, and the supernatant was called “supernatant”. The precipitated o-SWCNT-PEG-IgG/ProG-beads were washed twice with PB solution (40 μl) then re-dispersed in PB solution (40 μl). To elute the o-SWCNT-PEG-IgGs from the ProG-beads, 1 wt% SDBS solution (40 μl) was added to the o-SWCNT-PEG-IgG/ProG-beads in PB and sonicated with a tip-type sonicator (SONICS, VCX130) for 1 min. The ProG-beads were collected with a permanent magnet, and the 80 μl of supernatant containing o-SWCNT-PEG-IgG eluted from the beads was referred to as “elution”. Also 40 μl of “original” and “supernatant” were added to 1 wt% SDBS solution (40 μl) and sonicated to conform to “elution” in the preparation condition.

### NIR imaging

For *in vivo* imaging of mice, we used mice of the BALB/cAJcl-nu/nu line (CLEA Japan, Inc.). The mice were female and 6–8 weeks in age when the o-SWCNTs-PEG probe was injected. The *in vivo* NIR images were taken with the NIR imaging system (Shimadzu, SAI-1000) after ep-SWCNT-PEG probe administration from the tail vein (50 µg/animal). The images were excited at 980 nm (5 W) and exposed for 200 msec. Fluorescence was detected using a InGAaS camera with a filter at 1080 nm. All animal experimental protocols were approved by the Animal Care and Use Committee of AIST. All methods were carried out in accordance with the relevant guidelines and the regulations.

## Electronic supplementary material

Supporting Information

Video S1

Video S2

Video S3
