# Supplementary material for: Oxygen-doped carbon nanotubes for near-infrared fluorescent labels and imaging probes
Source: Sci Rep. 2018 Apr 19;8:6272. doi: 10.1038/s41598-018-24399-8 (PMC5908862; doi:10.1038/s41598-018-24399-8)
Supplement: Supplementary file 1 — Supporting Information [file 41598_2018_24399_MOESM1_ESM.pdf]

Supporting Information for

Oxygen-doped carbon nanotubes for near-infrared fluorescent labels and imaging probes

Yoko Iizumi,[1] Masako Yudasaka,[2] Jaeho Kim,[3] Hajime Sakakita,[3] Tsukasa Takeuchi[1,4] and Toshiya Okazaki[1,*]

[1]CNT-Application Research Center, National Institute of Advanced Industrial Science and Technology (AIST), 1-1-1 Higashi, Tsukuba 305-8565, Japan
[2]Nanomaterials Research Institute, National Institute of Advanced Industrial Science and Technology (AIST), 1-1-1 Higashi, Tsukuba 305-8565, Japan
[3]Electronics and Photonics Research Institute, National Institute of Advanced Industrial Science and Technology (AIST), 1-1-1 Umezono, Tsukuba 305-8568, Japan
[4]Shimadzu Corporation, 1-3 Kanda Nishiki-cho Chiyoda Tokyo 101-8448, Japan

E-mail: toshi.okazaki@aist.go.jp

The curve fitting of the observed PL spectrum

Figure S1 shows the observed PL spectrum after the oxidation process (black line) and the fitted curve by multiple Lorentzian functions (red line). The PL intensity ratio between eth-SWCNTs and ep-SWCNTs was 1:1.3 based on the spectral fitting result.

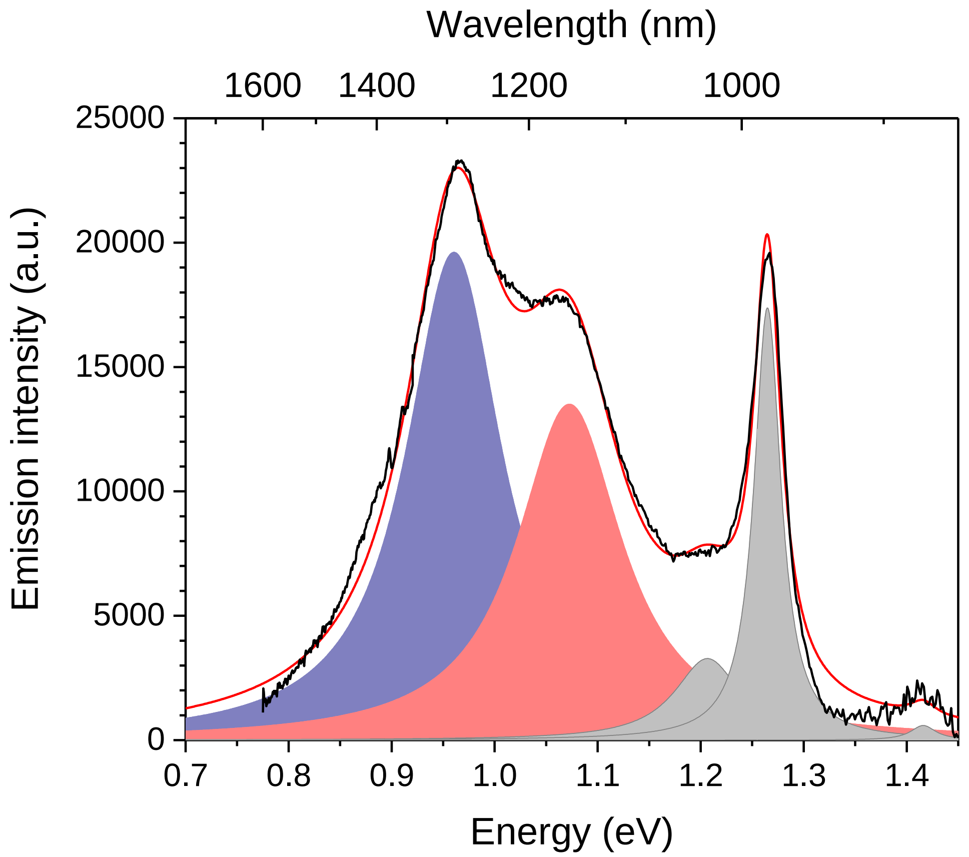

Figure S1. The observed PL spectrum obtained from SDBS-D_2O solution of SWCNTs after the UV irradiation for 90 sec. (black line) and the fitted curve by multiple Lorentzian functions (red line).

Table S1. Spectral data obtained by the curve fitting of the PL spectrum of o-SWCNTs with multiple Lorentzian functions (Figure S1).

Peak position (eV)	FWHM (eV)	Peak area	Assignments
0.96	0.11	3483	Epoxide-type (6, 5)
1.07	0.12	2637	Ether-type (6, 5)
1.27	0.032	869	Pristine (6, 5)
1.21	0.078	403	(7, 5)
1.42	0.030	28	(6, 4)

The oxidation of SWCNT thin films with various thickness

Since the UV light was irradiated to the SWCNT film from one direction in this synthesis experiment, the film thickness of the sample is important for efficient oxidation reaction. The oxidation reaction was conducted by using SWCNT thin film samples with various thickness. The thickness of CNT film was controlled by changing the amount of SWCNTs dispersed in ~10 mL of 1 wt% SDBS-D_2O (0.5 mg, 1.0 mg and 1.5 mg) (see Method). Figure S2 shows the PL spectra obtained from SDBS-D_2O solution of SWCNTs after the UV irradiation for 120 sec. Based on the curve fitting of the spectra with multi-functional Lorentz functions, the ratios of the peak area of ep-SWCNTs to the total area were 0.44, 0.41 and 0.29 for SWCNTs with the thickness of 0.37, 0.74 and 1.5 μm, respectively. The thickness of the films was measured with a laser microscope (Shimadzu, SFT-4500).

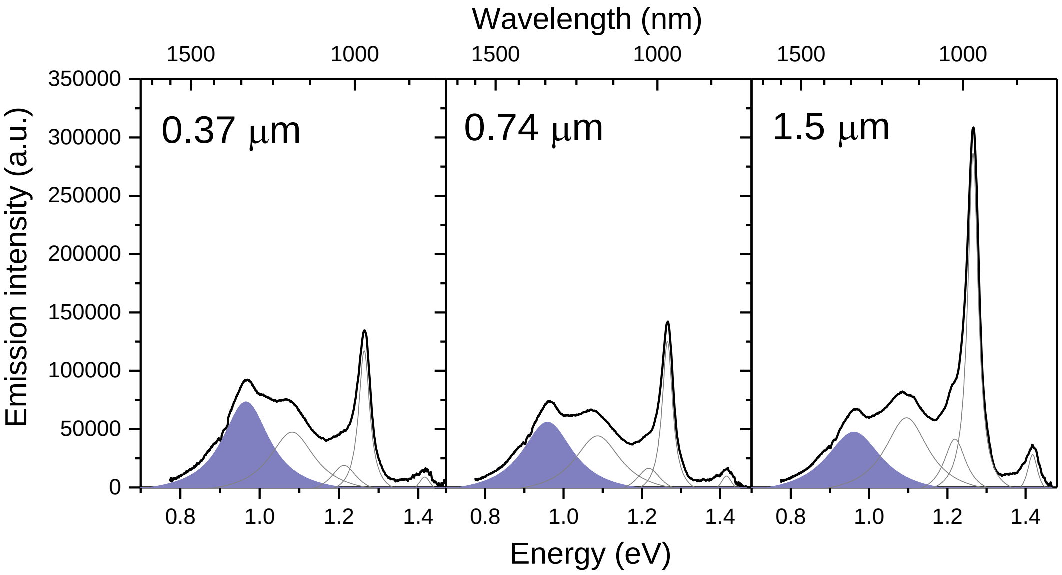

Figure S2. The observed PL spectra obtained from SDBS-D_2O solution of SWCNTs with various thickness.
